# A New Approach for Heparin Standardization: Combination of Scanning UV Spectroscopy, Nuclear Magnetic Resonance and Principal Component Analysis

**DOI:** 10.1371/journal.pone.0015970

**Published:** 2011-01-18

**Authors:** Marcelo A. Lima, Timothy R. Rudd, Eduardo H. C. de Farias, Lyvia F. Ebner, Tarsis F. Gesteira, Lauro M. de Souza, Aline Mendes, Carolina R. Córdula, João R. M. Martins, Debra Hoppensteadt, Jawed Fareed, Guilherme L. Sassaki, Edwin A. Yates, Ivarne L. S. Tersariol, Helena B. Nader

**Affiliations:** 1 Departamento de Bioquímica, Disciplina de Biologia Molecular, Universidade Federal de São Paulo, São Paulo, Brazil; 2 School of Biological Sciences, University of Liverpool, Liverpool, United Kingdom; 3 Department of Pathology, Loyola University Medical Center, Maywood, Illinois, United States of America; 4 Laboratório de Química de Carboidratos, Departamento de Bioquímica e Biologia Molecular, Universidade Federal do Paraná, Curitiba, Paraná, Brazil; Instituto Butantan, Brazil

## Abstract

The year 2007 was marked by widespread adverse clinical responses to heparin use, leading to a global recall of potentially affected heparin batches in 2008. Several analytical methods have since been developed to detect impurities in heparin preparations; however, many are costly and dependent on instrumentation with only limited accessibility. A method based on a simple UV-scanning assay, combined with principal component analysis (PCA), was developed to detect impurities, such as glycosaminoglycans, other complex polysaccharides and aromatic compounds, in heparin preparations. Results were confirmed by NMR spectroscopy. This approach provides an additional, sensitive tool to determine heparin purity and safety, even when NMR spectroscopy failed, requiring only standard laboratory equipment and computing facilities.

## Introduction

Heparin, a sulfated glycosaminoglycan (GAG) present in several mammalian and other vertebrate and invertebrate tissues [1], [2], [3], [4], has been an established anticoagulant drug for more than 60 years and is widely used for the prevention and control of thrombotic events owing to its interaction with a number of proteins of the blood clotting cascade, notably antithrombin and thrombin. It consists of a linear, highly sulfated polysaccharide chain comprising repeating disaccharide units of 1,4 *O*-linked α-L-iduronic or β-D-glucuronic acid, and α-D-glucosamine. The predominant substitution pattern comprises 2-*O*-sulfation of the iduronate residues and *N*- and 6-*O*-sulfation of the glucosamine residues [5], [6], [7]. The distribution of these sulfates in the polysaccharide chain provides heparin an average of 3.5 negative charges per disaccharide unit [8]. Other substitution patterns are also possible, including *N*-acetylation and 3-*O*-sulfation of the glucosamine, as well as non-sulfated iduronate and β-D-glucuronate, and 2-O-sulfation of glucuronate, providing considerable sequence heterogeneity [9] and making the structural elucidation of heparin challenging.

The pharmacological activity of heparin arises from its capability to bind and accelerate the activity of antithrombin (AT) [10], which considerably enhances the inhibition of coagulation factors Xa and IIa. The heparin anticoagulant effect can be directly correlated to the inhibition of factor Xa by AT [11], which catalyzes the conversion of prothrombin to thrombin, decreasing thrombin generation and, ultimately, the formation of a fibrin clot.

Pharmaceutical heparin is largely obtained from porcine intestinal mucosa by a multi-step extraction process that involves proteolysis, anion exchange chromatography or quaternary ammonium complexes, ethanol precipitation and bleaching. Throughout this process other naturally occurring GAGs, such as chondroitin (CS), dermatan (DS) and heparan sulfates (HS), as well as proteins and small molecules accompany heparin and, additionally, heparin may be modified [12]. The removal of these additional compounds incurs loss [13], which on an industrial scale is an undesirable outcome. Since this process is quite complex, it requires carefully executed procedures and effective quality control monitoring to avoid the co-purification of impurities and contaminating species.

Owing to the complex isolation and purification required, the issue of heparin impurities has long been recognized and was first brought up in 1955 when traces of phosphate were found in commercial heparins [14]. Ethylenediaminetetraacetic acid (EDTA) and histamine have also been identified in commercial heparin [15], [16], and the presence of EDTA has been associated with increased bleeding effects [15].

In 1989, nuclear magnetic resonance spectroscopy (NMR) was used to identify the presence of other GAGs in pharmaceutical heparins [17]. Dermatan sulfate (DS) was found to be the most common impurity comprising up to 10–15 percent of the polymer mixture. Again, in 2001, DS, sodium acetate and ethanol were identified as frequent impurities in heparin preparations [18]. Additionally, in early 1990's the outbreak of bovine spongiform encephalopathy (BSE) resulted in the exclusion of materials originating from ruminants for the production of heparin. Several methods involving molecular biology and immunochemical approaches were used to check the raw materials used by heparin manufacturers [19], [20]. However, none of these issues led to any reported adverse clinical responses, or resulted in significant number of deaths.

In 2008, significant numbers of adverse clinical responses associated with heparin use were first reported, leading to at least 149 deaths in the United States. Once again, using NMR and other analytical methods, the major contaminant present in heparin batches related to adverse clinical events was identified as an oversulfated chondroitin sulfate (OSCS) [21]. Several papers were then published regarding analytical approaches to assess heparin purity [13], [22], [23], [24], [25], [26]. However, many of these methods are expensive, time consuming and/or require access to sophisticated and costly instrumentation and facilities, making them difficult to use for producers and regulatory authorities in many locations.

Recently, the ability of circular dichroism (CD), a UV-based technique to differentiate GAGs effectively was demonstrated [5]. Differentiation was improved with the use of principal component analysis (PCA), a powerful statistical tool that allows the identification of patterns in any dataset and highlights their similarities and differences. This approach has been used to extract relationships from many forms of spectra and numerical data related to GAGs [5], [27], [28], [29].

In the present paper, we investigate the possibility of a simpler UV-based technique - scanning UV spectroscopy, combined with PCA, as a potential method for the assessment of heparin purity. The more technically demanding and expensive NMR technique was also employed as an external control of the effectiveness of the method.

## Results

### NMR confirmation of heparin purity

**Figure 1:**
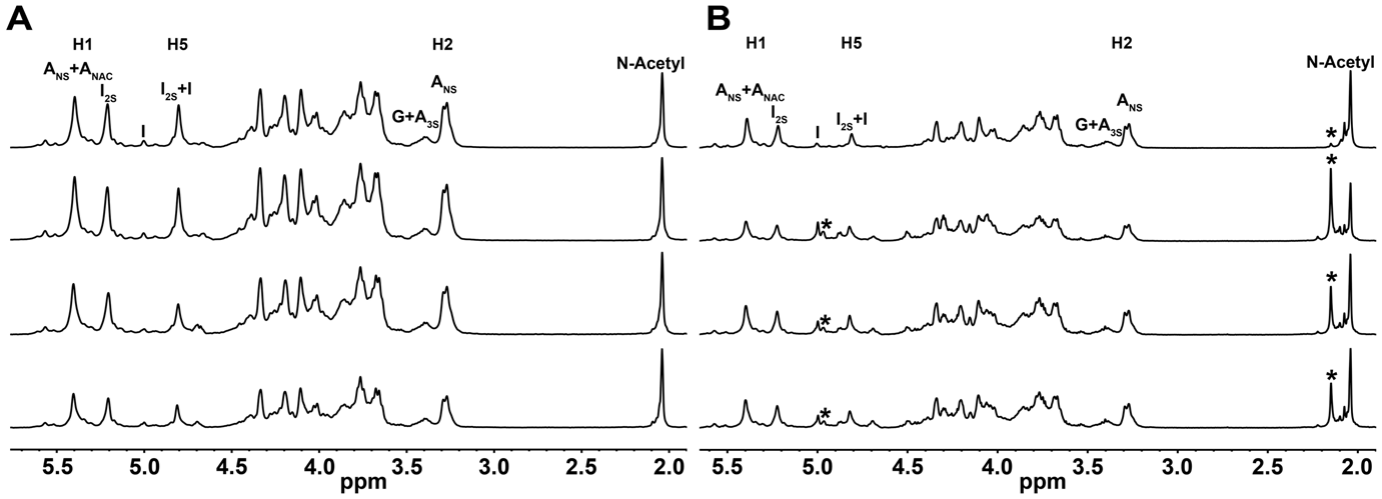
NMR analysis of heparin samples. (**A**) ^1^H NMR spectra of pure heparin samples. (**B**) ^1^H NMR spectra of contaminated heparin samples. From bottom to top samples a, b, c and d (**A**) and e, f, g and h (**B**). Signals due to the contaminants are highlighted by asterisks. A_NS_, 2-deoxy-2-sulfoamino-D-glucopyranose; A_3S_, 2-deoxy-3-O-sulfo-2-amino-D-glucopyranose; A_NAC_, 2-deoxy-2-acetylamino-D-glucopyranose; I_2S_, 2-O-sulfo-iduronic acid; G, glucuronic acid; I, iduronic acid.

^1^H NMR was performed according to the USP heparin monograph on 8 heparin samples in order to assess their purity to establish a controlled baseline for subsequent analysis. Special attention was given to the spectral region in which the *N*-acetyl group appears ∼2.00 p.p.m. Typical ^1^H NMR spectra of unfractionated heparins (UFHs a, b, c and d) are shown in Figure 1A. Major signals correspond to the trisulfated disaccharide (α-L-iduronate 2-*O*-sulfate 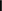
→4 α-D-glucosamine *N*,*O*-disulfate), which is the prevalent disaccharide repeating unit in heparin. No additional *N*-acetyl group signal was observed, leading to the conclusion that these preparations are free of other GAG species.

Figure 1B shows the presence of an additional signal, in the ^1^H NMR spectra of samples e, f, g and h, at 2.15 p.p.m, corresponding to *N*-acetyl groups distinct from that of heparin *N*-acetyl signals at 2.04 p.p.m and from those of DS at 2.08 p.p.m. This additional signal corresponds to the *N*-acetyl group of the previously described OSCS, which was shown to be the major contaminant present in heparin preparations related to adverse clinical responses [21]. Also, the ^1^H NMR spectra of the contaminated samples displayed strong signals, 7.4–7.8 p.p.m, consistent with aromatic compounds, which were further characterized as benzyl alcohol (Figure S1).

### Scanning UV spectroscopy is able to identify other GAG impurities in heparin samples

**Figure 2:**
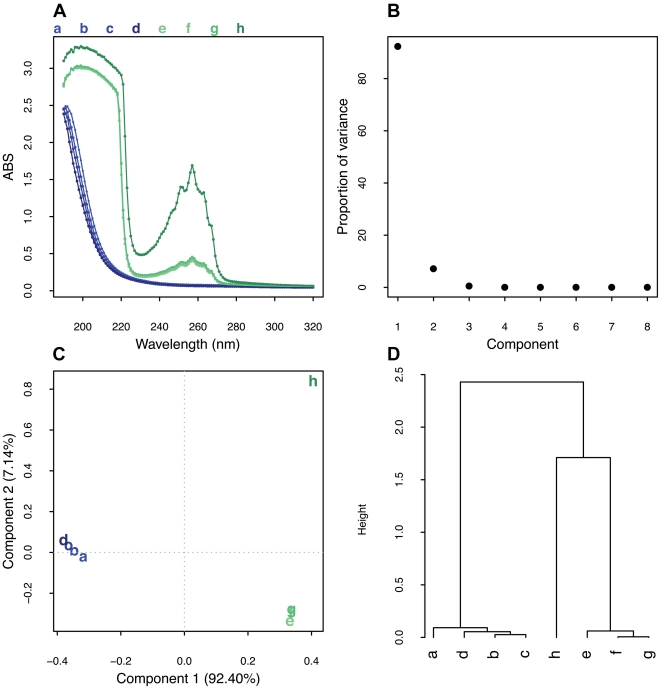
UV spectra, loading plot and Hierarchical cluster analysis of heparin samples. (**A**) Overlay of heparin samples scanning UV spectra. (**B**) Scree plot for PCA of the UV-scan spectra. (**C**) Component plot. (**D**) Hierarchical cluster analysis performed on the loading plot. Samples a–d represent pure heparin samples and e–h are the commercial contaminated heparins.

The features in the UV spectra [190–320 nm] of GAGs arise predominantly from electronic transitions in the carboxylate groups of the uronate residues and the *N*-acetyl chromophores in the glucosamine residues (n→π* and π→π * transitions), the carboxylate chromophore being responsible for the majority of the spectral features. Typical scanning UV spectra of samples containing carboxylic acid groups were observed with the highest peak around 190 nm, arising from the electronic transition occurring in the carboxylate chromophore of iduronate and glucuronate present in the GAG chains. Two distinct groups were easily identified; the pure samples (a, b, c and d) displayed only one sharp band from 190–210 nm while, on the other hand, the contaminated samples (e, f, g and h) displayed a much broader signal around 200–220 nm and an additional broad signal around 240–260 nm with a maxima at 257 nm (Figure 2A). This latter signal corresponds to electronic transitions arising from aromatic compounds being comparable to the UV scanning spectrum of benzyl alcohol [30].

The resulting UV spectra were next analyzed by PCA, in which the first two components described 99.54% of the variance (Figure 2B). The first principal component was sufficient to differentiate pure from contaminated samples, while the second principal component picked out the differences between the two groups (Figure 2C). The cluster analysis performed on the loading plot (Figure 2D) produced two distinct clusters, one comprising pure samples and the other containing contaminated samples, demonstrating that the analysis of scanning UV spectra by PCA is a powerful method to differentiate pure from impure heparin preparations.

### Scanning UV spectroscopy also differentiates other GAGs from heparin preparations

**Figure 3:**
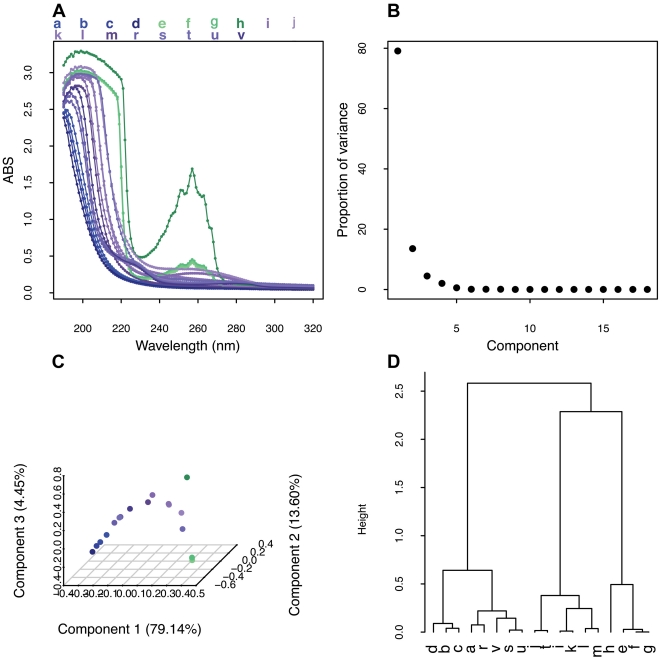
Multivariate analysis of UV spectra of heparins and other GAGs. (**A**) Overlay of GAGs scanning UV spectra. (**B**) Scree plot of components. (**C**) Plot of the first three components. (**D**) Hierarchical cluster analysis performed on the loading plot. Samples a–d represent pure heparin samples; e–h are the commercial contaminated heparins, i–k and t represent DS, C4S, C6S and HS respectively; l–m are SOSCS and IHC; r–s are heparin spiked with 5 and 10% of heparin-by product; u–v correspond to heparin spiked with 5 and 10% of HS.

In addition to identifying semi-synthetic contaminants, the method was able to detect the presence of other glycosaminoglycans impurities, including CS, DS, HS and heparin by-products (Figure 3A). The presence, in higher levels, of *N*-acetyl chromophores at slightly different chemical shift positions in the ^1^H spectra of other GAGs resulted in UV spectra which were distinct from that of heparin.

PCA analysis of these spectra revealed that the first three principal components described 97.19% of the variance, and these were used to differentiate the samples (Figure 3B). Component 1 was able to differentiate pure heparin samples from other GAGs and contaminated samples, while components 2 and 3 differentiated pure from impure and contaminated heparin (Figure 3C). The presence of semi-synthetic sulfated GAGs in contaminated heparin samples produced a unique spectrum, which was easily differentiated by PCA of the UV spectrum and shown by the cluster analysis (Figure 3D).

### Scanning UV spectroscopy detects the presence of non-mammalian polysaccharides in heparin preparations

**Figure 4:**
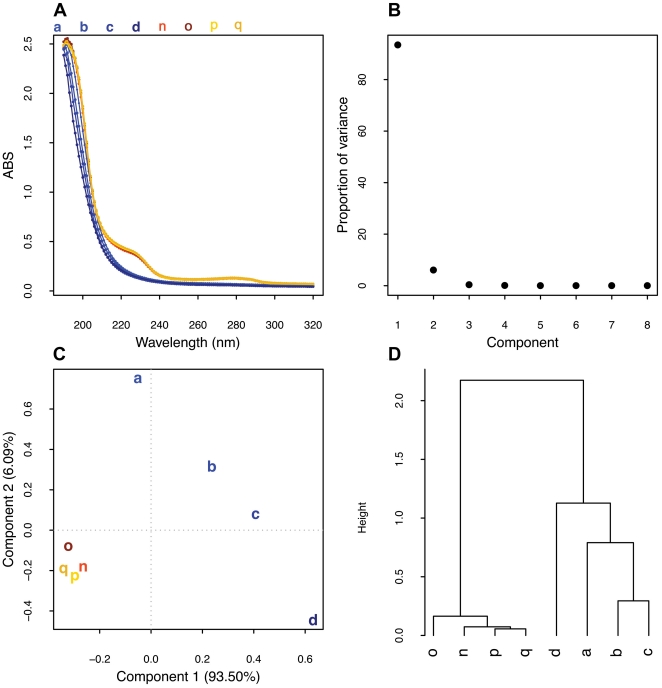
Multivariate analysis of UV spectra of heparin spiked with non-mammalian polysaccharides. (**A**) Overlay of heparin preparations scanning UV spectra. (**B**) Scree plot of components. (**C**) Component plot. (**D**) Hierarchical cluster analysis performed on the loading plot. Samples a–d represent pure heparin samples; n–p correspond to heparin spiked with 5 and 10% of SA respectively; g–h are heparin spiked with 5 and 10% of SG.

A variety of polysaccharides with charge properties, molecular weight and anticoagulant activities similar to heparin are well known. This led us to test whether scanning UV spectroscopy combined with PCA would be able to differentiate pure heparin from heparin that had been spiked with two non-mammalian polysaccharides. The presence of both contaminants produced two extra bands at 215–240 nm and 270–290 nm in the UV spectra (Figure 4A).

Further analysis of the UV spectra using PCA showed that the first two principal components described 99.59% of the variance present in the data set (Figure 4B). The component score plot for component 1 (Figure S2A) shows that this variance arises from differences in the electronic transition occurring mainly in the carboxylate groups, a fact that may arise from structural variability among heparin preparations. However, the component score plot for component 2, which accounts for only 6.09% of the variance, indicated that the differentiating features are the extra bands from 215–240 nm and 270–290 nm (Figure S2B). The two components were used to differentiate the samples (Figure 4C) and for the cluster analysis (Figure 4D).

### Combination of scanning UV spectroscopy and PCA detects as low as 0.1% contaminants in heparin samples

The method was able to successfully detect impurities and contaminants in heparin. The ability of the method to identify only traces of contaminants was then assessed.

**Figure 5:**
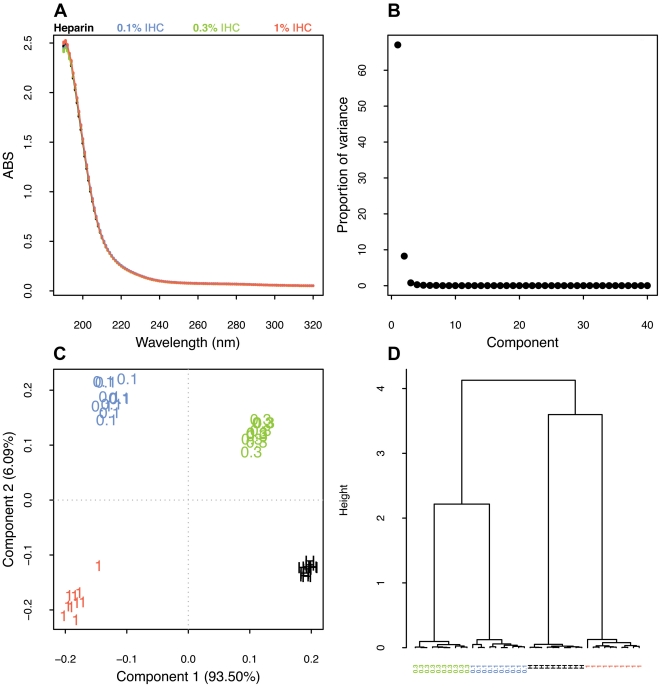
UV spectra, loading plot and Hierarchical cluster analysis of heparin preparations. (**A**) Overlay of heparin preparations UV-scan spectra. (**B**) Scree plot of components. (**C**) Component plot. (**D**) Hierarchical cluster analysis performed on the loading plot.

In order to address this question, a pure heparin sample was spiked with 0.1, 0.3, 1, 1.3, 1.6, 2, 3 and 5% (w/w) of the isolated heparin contaminant (IHC) and their respective scanning UV spectra recorded. PCA analysis of the UV spectra revealed that this approach was able to distinguish pure heparin from the spiked heparin preparations (Figure S3A and B), yet this differentiation was questionable for contaminant levels lower than 1%, since spectral fluctuation could mask the results. For this reason, multiple spectra were recorded for each sample (Figure 5A). The resulting UV spectra were next analyzed by PCA, in which the first two components described 99.59% of the variance (Figure 5B). Indeed, as shown by the PCA analysis, there was significant spectral fluctuation, however, the variability occured within the sample groups as shown by the loading plot (Figure 5C) and cluster analysis (Figure 5D).

## Discussion

The issue of heparin impurities has been well-known since 1955, when the first paper was published regarding the identification of phosphate in heparin preparations [14]. Subsequently, mixtures of glycosaminoglycans and other organic and inorganic impurities were found in pharmaceutical heparin preparations [15], [16], [17], [18]. With the outbreak of BSE this matter resurfaced, however, none of these problems led to a major heparin recall across the globe.

In 2008, health authorities in the United States received several alerts regarding acute hypersensitivity reactions due to the use of heparin in patients undergoing dialysis. These reports led to a major recall, first in the USA and then Europe, of heparin batches, heparin coated medical devices, and lock flush-injections that used heparin as the active pharmaceutical ingredient.

By June of the same year Guerrini and colleagues had identified and characterized the major contaminant in heparin batches as an oversulfated chrondroitin sulfate (OSCS) [21], a compound that does not exist naturally, which led to the conclusion that the contamination was deliberate. Subsequently, several other groups published papers on the same subject using a wide variety of methods [23], [24], [25], [26], [31]. More recently, the contaminant was claimed to compromise not only OSCS but a mixture of other chemically sulfated GAGs, probably arising from waste generated throughout the heparin production process [22]. Also, the mechanism in which the OSCS contaminant caused the anaphylactoid responses was characterized [32], and recently how these semi-synthetic GAGs activate the complement system and how they affect GAG-dependent cell signaling pathways have also been investigated [33].

The methods capable of identifying impurities and suitable for the assessment of heparin purity and safety are often based on NMR, which is a very time consuming and expensive technique since it relies on technically complicated procedures using expensive machines that are not available in many laboratories, a fact that makes it difficult for all but the best equipped laboratories to monitor heparin preparations on a large scale.

Moreover, the presence of significant amounts of DS in drugs constituted by GAG mixtures raises the limit of detection of OSCS at 500MHz from 0.05% to 0.2% [12]. In addition, peaks can shift depending on the counter ion present, i.e. calcium versus sodium, as well as the exact sulfation pattern of the heparin and/or the contaminants [34], despite the fact that NMR based methods were developed to specifically monitor the presence of oversulfated GAGs species, which exclude a great number of non-*N*-acetylated potential contaminants with charge properties, molecular weight and anticoagulant activities similar to heparin.

Recently, the combination of circular dichroism, an UV-based spectroscopy, with multivariate analysis methods have allowed differentiation of low molecular weight heparins obtained by different methods of production, as well as naturally occurring GAGs [29]. This showed in principle that relatively simple methods were able to differentiate between members of this class of highly complex carbohydrate.

**Figure 6:**
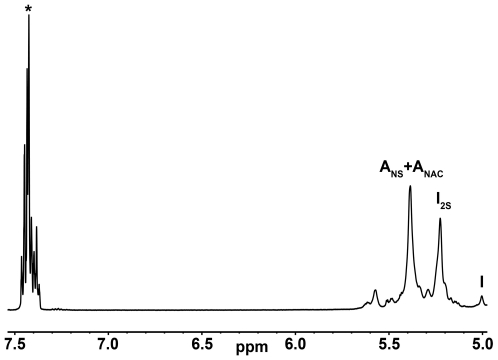
Selected ^1^H NMR region showing the benzyl alcohol signals found on the contaminated heparin samples spectra. Signals due to benzyl alcohol are highlighted by asterisks. A_NAC_, 2-deoxy-2-acetylamino-D-glucopyranose; A_NS_, 2-deoxy-2-sulfoamino-D-glucopyranose; I_2S_, 2-O-sulfo-iduronic acid; I, iduronic acid.

In the present paper, scanning UV spectroscopy - a very simple technique requiring only a standard scanning UV spectrophotometer, in combination with PCA -, was used to assess heparin purity on a series of samples whose purity had been confirmed previously by ^1^H NMR. The method was also capable of distinguishing pure from commercial contaminated heparin samples and heparin spiked with other GAGs, as well as non-*N*-acetylated polysaccharides, which ^1^H NMR have failed to identify [12], [31]. This differentiation was accomplished by virtue of the carboxylate chromophore of iduronate and glucuronate in the GAGs and the presence of *N*-acetyl groups, in higher levels and in slightly different environments in CS, DS, and HS. Other chromophores, such as the carboxylate groups in non-GAG polysaccharides as sodium alginate and pyruvate in sulfated galactans provided additional features to the UV-spectra. UV spectroscopy is also highly sensitive to the electronic transitions that occur within protein and aromatic impurities; an example of the latter are the signals present on the UV and ^1^H NMR spectra of the contaminated heparin samples (Figure 2A and 6), being them further identified as belonging to benzyl alcohol.

Benzyl alcohol, a bacteriostatic agent found in many parental preparations, has been associated with adverse clinical events [35], [36], for this reason the use of parental preparations containing such preservative agents is restricted. The effect of benzyl alcohol on vascular endothelial cells (VEC) was evaluated, employing a cell viability assay and revealing that benzyl alcohol is highly toxic to VEC (Figure S4); therefore, together with the fact that the latest bulletin regarding anticoagulant heparin solution states that it contains no antimicrobial agents [37], specific guidelines regarding the presence of this compound on heparin vials should be readdressed since.

The heparin contamination crisis reinforced the idea that the purity and relative uniformity of complex mixtures such as heparin requires the use of multiple, orthogonal analytical techniques [38]. However the adoption of the currently available techniques by heparin manufacturers will cause increased production costs and, ultimately, higher prices for the final product, therefore the search for cheaper and more versatile analytical approaches such as that presented here is of great importance. Taken together, the results showed that this simple approach may be used as an additional tool to verify heparin purity and safety. Furthermore, this approach allows the establishment of a database of standard heparins against which any given sample could be cross-checked, providing the level of similarity among them and highlighting their similarities and dissimilarities (presence of contaminants), besides the determination of whether a heparin sample should be subjected to the full quality assessment by highly sophisticated, modern and expensive spectroscopic methods.

## Materials and Methods

### Polysaccharides

Contaminated heparin samples were the recalled heparin lots withdrawn from the clinical use in the Hemodialysis Unit from the Loyola University Hospital in Maywood, IL or purchased from the Brazilian market. Semi-synthetic (SOSCS) and isolated heparin contaminant (IHC) were prepared as previously described [25]. Pure heparin samples were a gift from Dr. Valentina Baigorria (Kim Master Produtos Químicos Ltd, Brazil) and Gentium SpA (Villa Guardia (CO), Italy). The GAGs and polysaccharides chondroitin 4-sulfate (C4S), chondroitin 6-sulfate (C6S), dermatan sulfate (DS), and sodium alginate (SA) were purchased from Sigma-Aldrich (St. Louis, MO). Heparan sulfate (HS) and sulfated galactans (SG) were purified as previously described [39], [40]. These compounds, including the contaminants, can be distinguished by their electrophoretical mobility in agarose gel (Figure S5A and B) assessed as previously described [Reference S1].

### Nuclear Magnetic Resonance

^1^H NMR was performed as described in the USP unfracionated heparin (UFH) monographs with residual water signal suppression [41]. Briefly, samples of 20 mg/ml in deuterium oxide (99,9%, Cambridge Isotope Laboratories Inc, Andover, MA, USA) were used for the acquisition of a free induction decay (FID) using 16 scans, a 90° pulse and 20 s delay at 25°C in a Bruker DRX-500 spectrometer.

### Scanning UV spectroscopy

Scanning UV spectroscopy was performed on Perkin-Elmer Lambda 25 UV/VIS spectrometer. Samples of 1 mg/ml in water were scanned from 190 to 320 nm with 1 nm resolution at 120 nm/min. The resulting spectra were saved as ASC files for subsequent analysis using PCA.

### Factor Analysis

Factor analysis, of which principal component analysis is an example, is used to uncover the latent structure (dimensionality) of a complex data set and encapsulate the crucial information, while eliminating noise. PCA performs the optimum co-ordinate rotation to align the axes so that the variance within the data is maximized. This transforms a set of previously correlated variables into a set of uncorrelated ones, which are linear combinations of the original variables. The linear combination that extracts the maximum variance from the data is termed the principal component. Once this is found, this is removed and the process repeated to identify the next principal component. This continues until all the variance in the data has been explained (in practice, this is not achieved because of residual noise and the process is terminated).

### Multivariate Analysis

PCA and Cluster analysis were conducted using the software R: A Language and Environment for Statistical Computing (R Foundation for Statistical Computing, Viena, Austria. http://cran.r-project.org/), with prior mean centering.

### Definitions

*Components* represent the underlying dimensions that summarize or account for the original set of observed data. *Component loadings* are the correlation coefficients between variables and factors. The squared factor loadings indicate the percentage of the variance in the original variable that is explained by a factor. *Component scores* are a composite measure created for each observation on each factor extracted in the factor analysis.

## Supporting Information

Figure S1 Aromatic component identification via NMR spectroscopy. Bottom to top; Selected 1H NMR spectrum of Benzyl Alcohol and contaminated heparin sample. Note the signal correspondence on both spectra. A_NS_, 2-deoxy-2-sulfoamino-D-glucopyranose, I_2S_, 2-O-sulfo-iduronic acid.
(TIF)

Figure S2 Component score plot of heparin spiked with non-mammalian (sodium alginate and sulfated galactan) polysaccharides. (**A**) Component 1 score plot. (**B**) Component 2 score plot.
(TIF)

Figure S3 Loading plot and Hierarchical cluster analysis of heparin preparations. (**A**) Plot of the first two components. (**B**) Hierarchical cluster analysis performed on the loading plot. UFH, Unfracionated heparin; SUFH, Spiked unfracionated heparin.
(TIF)

Figure S4 Benzyl Alcohol Cytotoxicity. Cytotoxicity was determined using the 3-(4,5-dimethylthiazol-2-yl)-2,5-diphenyltetrazoliumbromide (MTT) assay. For this assay, 10^5^ vascular endothelial cells were seeded in 96-well plates and cultured for 2 days. The medium was removed and fresh medium containing 10% FBS and different amounts of Benzyl Alcohol or only fresh medium (control) were added being the cells maintained for 24 hours (37°C, 5% CO_2_). Afterwards, the cells were washed with PBS and serum-free medium containing MTT (0.5mg/mL) was added. After 2 hours of incubation, isopropanol extraction was performed and the absorbance measured at 570 nm with an ELISA reader (ELx800 BioTek Instruments, Winooski, VT).
(TIF)

Figure S5 Agarose gel electrophoresis in different buffer systems [Reference S1]. Briefly, aliquots (5 µg) of sGAGs were applied to a 0.6% agarose gel and ran for 1 h at 100 V. The sGAGs in the gel were fixed with 0.1% *N*-cetyl-*N*,*N*,*N*-trimethylammonium bromide solution. After 2 h, the gel was dried and stained with 0.1% toluidine blue in acetic acid/ethanol/water (0.1∶5∶5, v/v). (**A**) 0.05M 1,3-diaminopropane acetate buffer pH 9. (**B**) Discontinuous barium acetate/1,3-diaminopropane acetate buffer system. CUFH, contaminated unfracionated heparin; PUFH, porcine unfracionated heparin; BUFH, bovine unfracionated heparin; sGAG, sulfated glycosaminoglycans; SOSCS, semi-synthetic oversulfated chrondroitin sulfate; ICH, isolated heparin contaminant; Hep, heparin; F, fast moving component; I, intermediate moving component; S, slow moving component; Org., origin; CS, chrondroitin sulfate; DS, dermatan sulfate; HS, heparan sulfate.
(TIF)

References S1 (DOC)
