# Supplementary figures and images for: A New Approach for Heparin Standardization: Combination of Scanning UV Spectroscopy, Nuclear Magnetic Resonance and Principal Component Analysis
Source: PLoS One. 2011 Jan 18;6(1):e15970. doi: 10.1371/journal.pone.0015970 (PMC3022730; doi:10.1371/journal.pone.0015970)

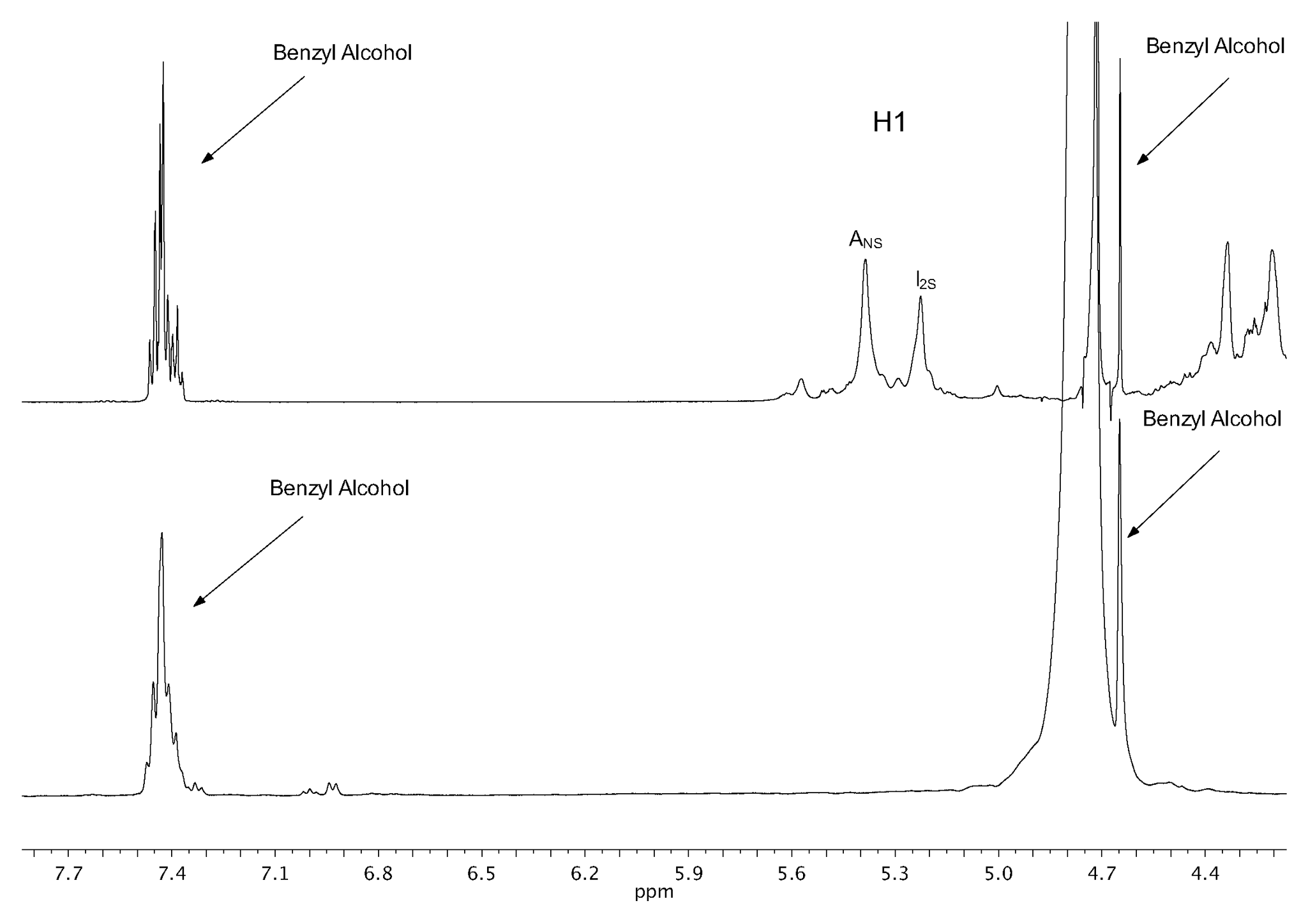

Supplement: Figure S1 — Aromatic component identification via NMR spectroscopy. Bottom to top; Selected 1H NMR spectrum of Benzyl Alcohol and contaminated heparin sample. Note the signal correspondence on both spectra. ANS, 2-deoxy-2-sulfoamino-D-glucopyranose, I2S, 2-O-sulfo-iduronic acid. (TIF) [file pone.0015970.s001.tif]

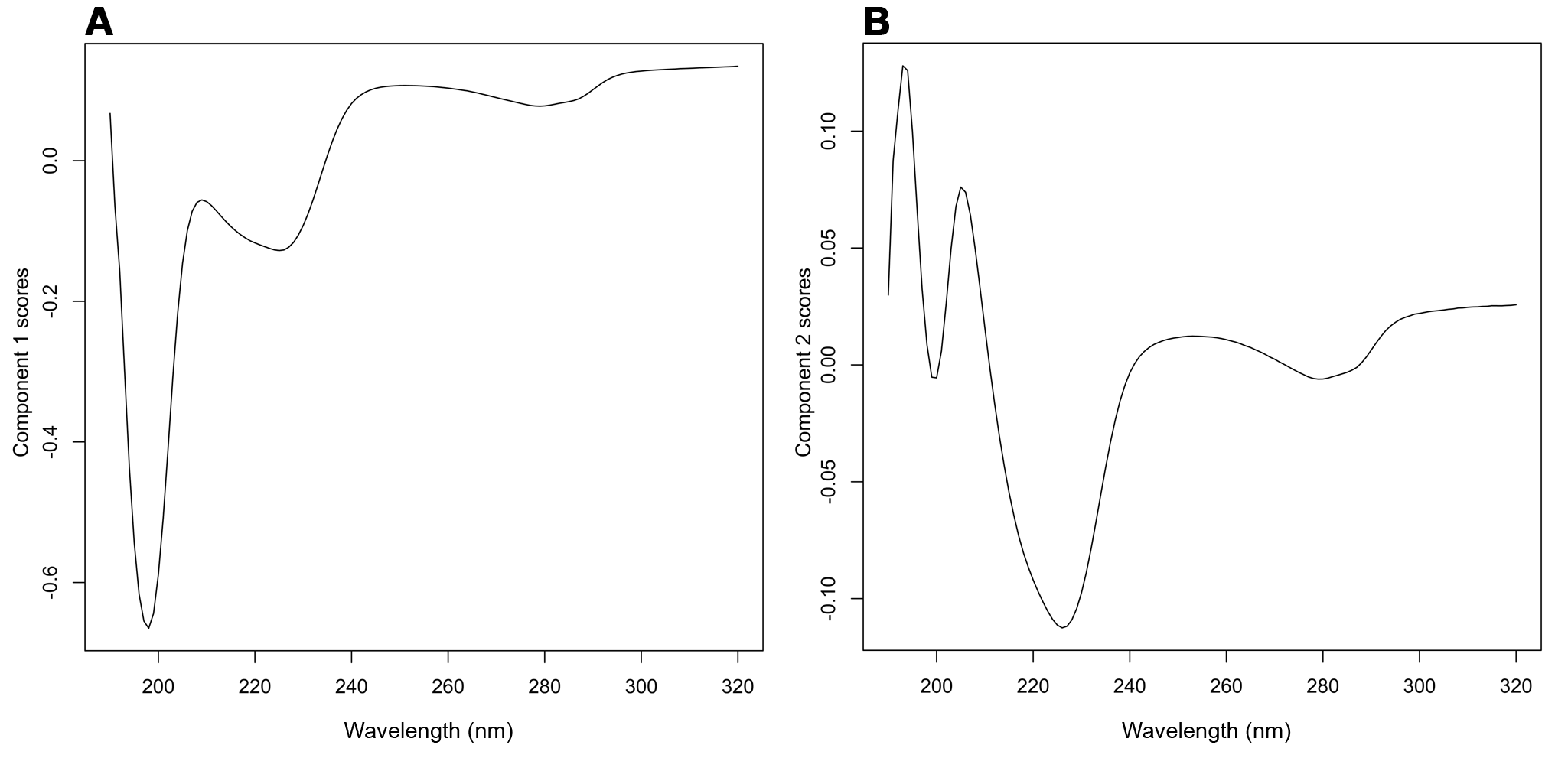

Supplement: Figure S2 — Component score plot of heparin spiked with non-mammalian (sodium alginate and sulfated galactan) polysaccharides. (A) Component 1 score plot. (B) Component 2 score plot. (TIF) [file pone.0015970.s002.tif]

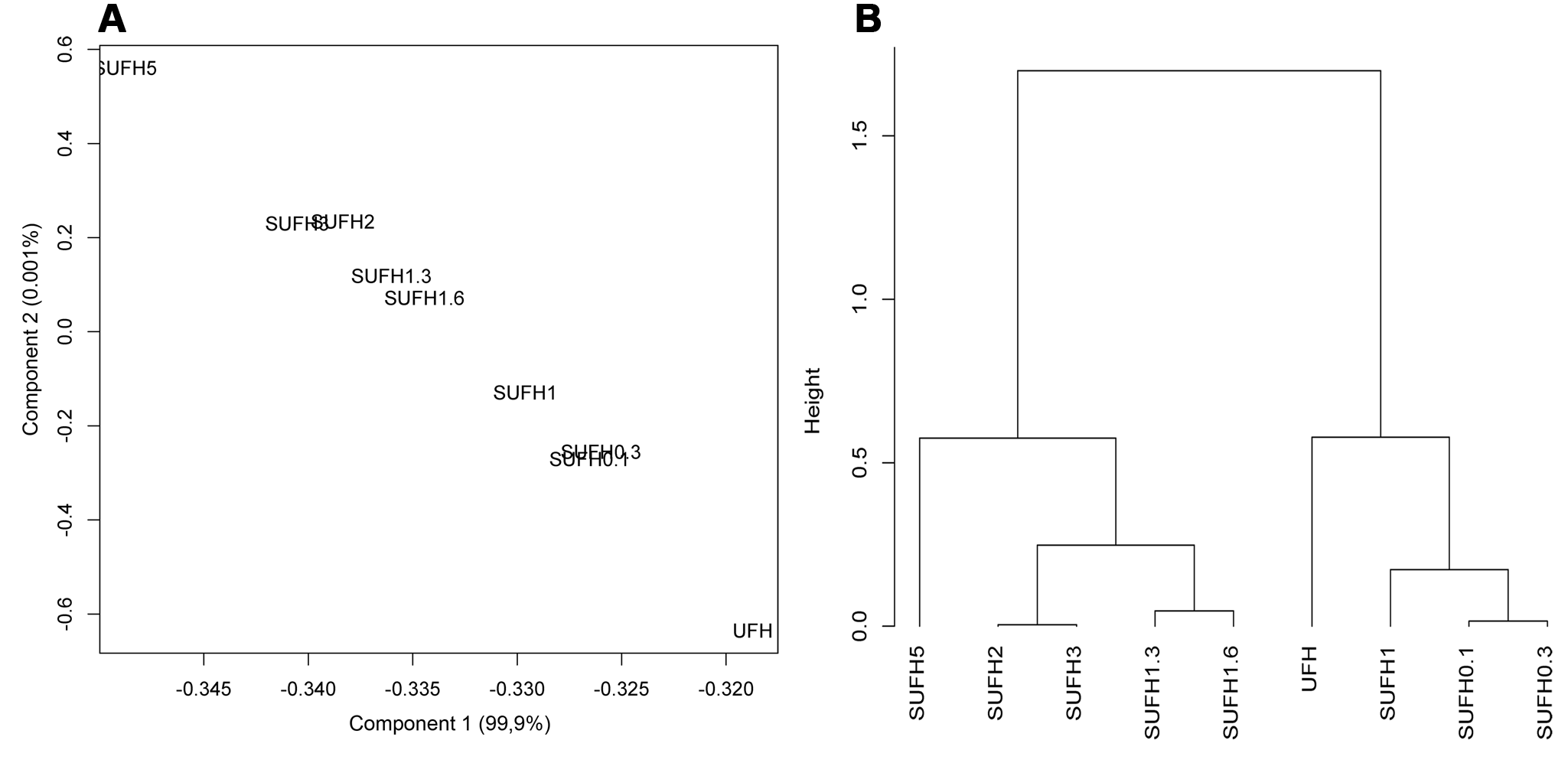

Supplement: Figure S3 — Loading plot and Hierarchical cluster analysis of heparin preparations. (A) Plot of the first two components. (B) Hierarchical cluster analysis performed on the loading plot. UFH, Unfracionated heparin; SUFH, Spiked unfracionated heparin. (TIF) [file pone.0015970.s003.tif]

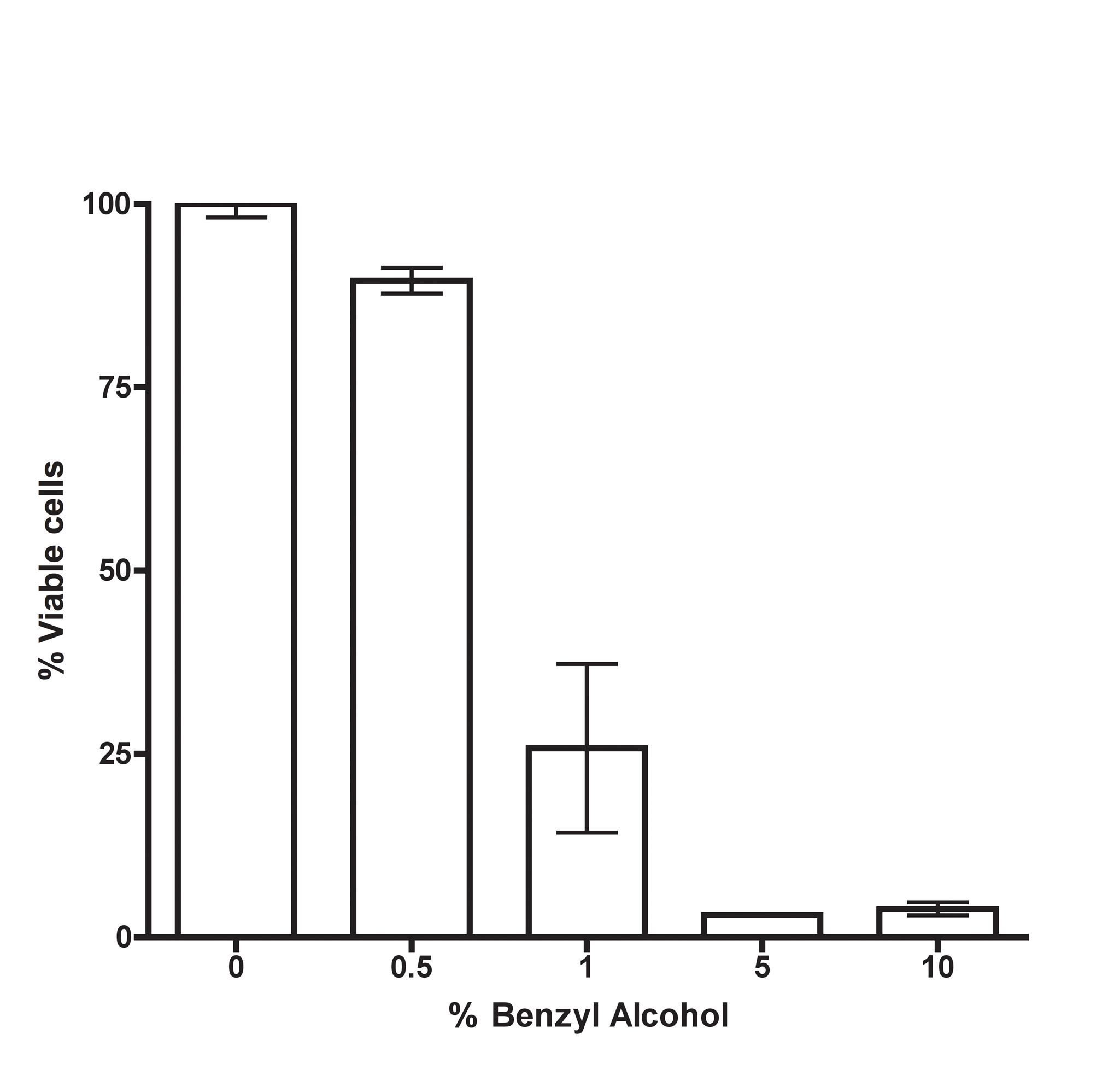

Supplement: Figure S4 — Benzyl Alcohol Cytotoxicity. Cytotoxicity was determined using the 3-(4,5-dimethylthiazol-2-yl)-2,5-diphenyltetrazoliumbromide (MTT) assay. For this assay, 105 vascular endothelial cells were seeded in 96-well plates and cultured for 2 days. The medium was removed and fresh medium containing 10% FBS and different amounts of Benzyl Alcohol or only fresh medium (control) were added being the cells maintained for 24 hours (37°C, 5% CO2). Afterwards, the cells were washed with PBS and serum-free medium containing MTT (0.5mg/mL) was added. After 2 hours of incubation, isopropanol extraction was performed and the absorbance measured at 570 nm with an ELISA reader (ELx800 BioTek Instruments, Winooski, VT). (TIF) [file pone.0015970.s004.tif]

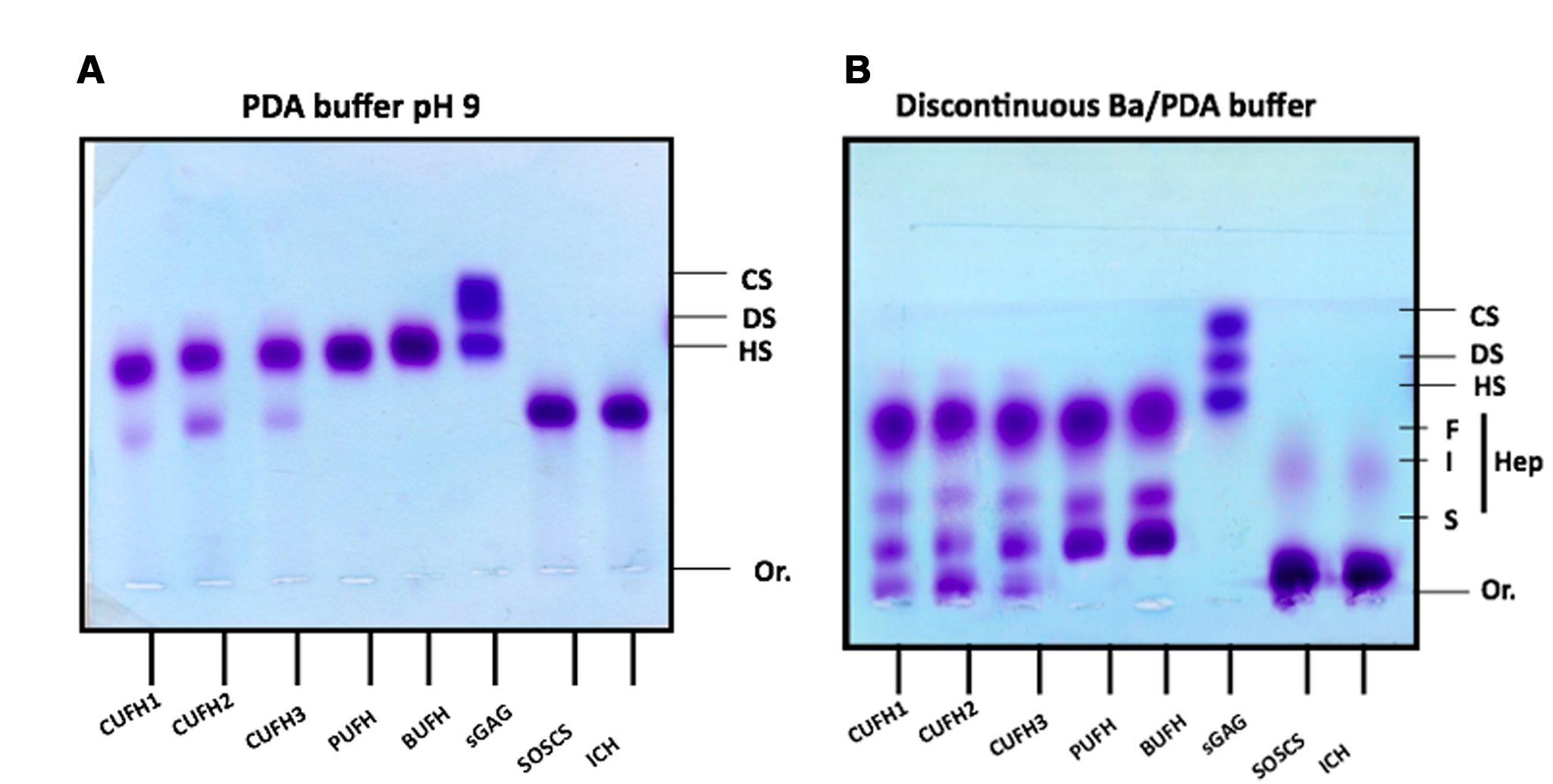

Supplement: Figure S5 — Agarose gel electrophoresis in different buffer systems [Reference S1]. Briefly, aliquots (5 µg) of sGAGs were applied to a 0.6% agarose gel and ran for 1 h at 100 V. The sGAGs in the gel were fixed with 0.1% N-cetyl-N,N,N-trimethylammonium bromide solution. After 2 h, the gel was dried and stained with 0.1% toluidine blue in acetic acid/ethanol/water (0.1∶5∶5, v/v). (A) 0.05M 1,3-diaminopropane acetate buffer pH 9. (B) Discontinuous barium acetate/1,3-diaminopropane acetate buffer system. CUFH, contaminated unfracionated heparin; PUFH, porcine unfracionated heparin; BUFH, bovine unfracionated heparin; sGAG, sulfated glycosaminoglycans; SOSCS, semi-synthetic oversulfated chrondroitin sulfate; ICH, isolated heparin contaminant; Hep, heparin; F, fast moving component; I, intermediate moving component; S, slow moving component; Org., origin; CS, chrondroitin sulfate; DS, dermatan sulfate; HS, heparan sulfate. (TIF) [file pone.0015970.s005.tif]
