# Supplementary material for: A New Approach for Heparin Standardization: Combination of Scanning UV Spectroscopy, Nuclear Magnetic Resonance and Principal Component Analysis
Source: PLoS One. 2011 Jan 18;6(1):e15970. doi: 10.1371/journal.pone.0015970 (PMC3022730; doi:10.1371/journal.pone.0015970)
Supplement: References S1 — (DOC) [file pone.0015970.s006.doc]

# Supporting Reference

S1. Bianchini P, Nader HB, Takahashi HK, Osima B, Straus AH, et al. (1980) Fractionation and Identification of Heparin and Other Acidic Mucopolysaccharides by a New Discontinuous Electrophoretic Method. Journal of Chromatography 196: 455-462.
